# Photo-Induced Super-hydrophilic Thin Films on Quartz Glass by UV Irradiation of Precursor Films Involving a Ti(IV) Complex at Room Temperature

**DOI:** 10.3390/ma12030348

**Published:** 2019-01-23

**Authors:** Hsiang-Jung Wu, Kota Tanabe, Hiroki Nagai, Mitsunobu Sato

**Affiliations:** 1 Applied Chemistry and Chemical Engineering Program, Graduate School, Kogakuin University of Technology and Engineering, 2665-1 Nakano, Hachioji, Tokyo 192-0015, Japan; bd16001@ns.kogakuin.ac.jp; 2 Department of Applied Physics, School of Advanced Engineering, Kogakuin University of Technology and Engineering, 2665-1 Nakano, Hachioji, Tokyo 192-0015, Japan; s415028@ns.kogakuin.ac.jp (K.T.); nagai@cc.kogakuin.ac.jp (H.N.)

**Keywords:** molecular precursor, metal complex, Amorphous, super-hydrophilicity, transparent thin film

## Abstract

Photo-induced super-hydrophilic thin films were fabricated on a quartz glass substrate by ultraviolet (UV) irradiation of a molecular precursor film at room temperature. A molecular precursor film exhibiting high solubility to both ethanol and water was obtained by spin-coating a solution involving a Ti(IV) complex; this complex was prepared by the reaction of Ti(IV) alkoxide with butylammonium hydrogen oxalate and hydrogen peroxide in ethanol. Transparent and well-adhered amorphous thin films of 160–170 nm thickness were obtained by weak UV irradiation (4 mW·cm^−2^ at 254 nm) of the precursor films for over 4 h at room temperature. The resultant thin films exhibiting low refractive indices of 1.78–1.79 were mechanically robust and water-insoluble. The chemical components of the thin films were examined by means of Fourier transform-infrared (FT-IR) and X-ray photoelectron spectroscopy (XPS) spectra, focusing on the presence of the original ligands. The super-hydrophilic properties (evaluated based on the water contact angles on the surfaces) of the thin films after being kept in a dark condition overnight emerged when the aforementioned UV-light irradiation was performed for 10 min. It was additionally clarified that the super-hydrophilicity can be photo-induced repeatedly by UV irradiation for 10 min (indicated by a contact angle smaller than 4°) even after the hydrophilic level of the thin films had once been lowered by being in a dark condition for 4 h.

## 1. Introduction

A hydrophilic surface effectively imparts self-cleaning and anti-fogging properties to various products such as windows, mirrors, and walls. In order to impart these properties to material surfaces, numerous methods have been investigated involving surface coating by TiO_2_ [1,2,3,4], WO_3_ composite [5], and polydimethylsiloxane [6].

Among these methods, TiO_2_ coating exhibits potential owing to the natural abundance, chemical stability, and non-toxic properties of the material [7,8]. The photocatalytic activity of TiO_2_ is higher with a crystal structure of anatase than that of rutile [9]. Moreover, superhydrophilicity of TiO_2_ is induced by surface oxygen vacancy, whereby surface oxygen vacancy is more easily to form by photo-generated electron-hole pairs in anatase than rutile [10]. Nagai et al. reported the fabrication of an O-deficient rutile thin film on a quartz glass substrate by heat treating a precursor film involving Ti(IV) complexes, by means of the molecular precursor method (MPM) [11]. The contact angles of 1.0-μL water droplets on the O-deficient rutile thin film were 13° ± 1° and 5° ± 1° by irradiating of visible- and UV light, respectively, indicating the photo-induced hydrophilicity.

The MPM is focused on the design of metal complex salts, which can dissolve into various solvents and form the corresponding genuine solutions [12]. The precursor solutions involving discrete molecular-weight metal complexes can be applied to various substrates through spin-coating [11,13] and spray-coating [14]. Generally, the coated precursor film that is spread homogenously on a substrate can be converted to metal or metal oxide thin films depending on the heat treatment parameters. Recently, the fabrication of a patterned Cu_2_O thin film exhibiting remarkable *p*-type nature, through UV-irradiation of a precursor film involving Cu(II) complexes was achieved by our group. The Cu(II) complexes involved in the precursor film were successfully converted to a crystalline Cu_2_O thin film by using UV-light from a germicidal lamp, at room temperature [15]. The environmental-friendly coating under atmospheric conditions at room temperature is favorable for obtaining functional thin films on glass substrates.

In this study, we fabricated a photo-induced hydrophilic thin film through UV-irradiation of a molecular precursor film involving a Ti(IV) complex salt spin-coated on a quartz glass substrate. Here, we report the fabrication and characterization of non-crystalline and transparent thin films exhibiting photo-induced super-hydrophilic property.

## 2. Materials and Methods

### 2.1. Materials

The chemicals used in this study were of analytical grade. Oxalic acid dihydrate and 2-propanol were purchased from Kanto Chemical Co., Inc. (Tokyo, Japan). Titanium tetraisopropoxide and butylamine were purchased from Wako Pure Chemical Industries, Ltd. (Osaka, Japan). Purified water was purchased from Kyoei Co., Ltd. (Tokyo, Japan). Hydrogen peroxide (31%) was purchased from Santoku Chemical Industries, Ltd. (Tokyo, Japan). Ethanol was purchased from Ueno Chemical Industries, Ltd. (Osaka, Japan) and was dried over 4A molecular sieves prior to use. The other chemicals were used without further purification. The ultraviolet germicidal lamp (GL-15) used for UV irradiation was purchased from Panasonic Co. (Osaka, Japan). The quartz glass substrate was purchased from Akishima-Glass Inc. (Okayama, Japan). The quartz glass substrate of dimensions 20 mm × 20 mm × 1.1 mm was washed in water with a detergent for 30 min through sonicated stirring. These glass substrates were rinsed in 2-propanol for 30 min using sonicated stirring and then dried in a drying oven at 70 °C.

### 2.2. Preparation of Precursor Solution Involving Ti(IV) Complex

A precursor solution was prepared according to our previous work [16]. A powder of butylammonium hydrogen oxalate hemihydrate was first prepared by mixing 11.4 g (156 mmol) of butylamine with 19.6 g (156 mmol) of oxalic acid in ethanol and then refluxed for 1 h. After cooling to room temperature, the white powder was collected by filtration and dried overnight.

A solution was prepared by mixing 1.13 g (3.96 mmol) of Ti(IV) tetraisopropoxide with 1.37 g (7.92 mmol) of butylammonium hydrogen oxalate hemihydrate in ethanol and then refluxed for 3 h. Then, 0.44 g (3.96 mmol) of hydrogen peroxide (31%) was added to the solution after cooling to room temperature; the resulting solution was refluxed for 0.5 h. The obtained solution **S**, in which the total concentration of Ti^4+^ was 0.4 mmol·g^−1^, was used as the precursor solution.

### 2.3. Fabrication of Precursor Film **F_0_** and Ultraviolet (UV)-Irradiated Thin Films **F_x_**

One hundred microliters of **S** were dropped on a quartz glass substrate of dimensions 20 mm × 20 mm using a micro-pipette. The precursor films on the quartz glass substrate were then formed using a spin-coating method with a double-step mode (1st: 500 rpm for 5 s and 2nd: 2000 rpm for 30 s). The precursor film (denoted as **F_0_**) was obtained by pre-heating the spin-coated substrates in a drying oven (ADVANTEC, Tokyo, Japan) at 70 °C for 10 min. The precursor films were irradiated with UV light of 254 nm (with a corresponding intensity of 4 mW·cm^−2^) for 1–16 h under 40–60% humidity in a clean bench. The substrate surface temperature during the UV-irradiation (measured using a digital thermocouple) was 30–40 °C. The UV-irradiated films on the quartz glass substrate are denoted as **F_x_** (x = 1, 2, 4, 8, and 16), where x indicates the UV-irradiation time (h).

In addition, a 365 nm black light lamp (intensity: 5.3 mW·cm^−2^) was investigated and UV-irradiate to **F_0_** for 16 h. However, the UV-irradiated film was completely dissolved by water, after contact with water.

### 2.4. Characterization of Precursor Solution **S**, Precursor Film **F_0_**, and UV-Irradiated Films **F_x_**

#### 2.4.1. Optical Characterization of **S**, **F_0_**, and **F_x_**

The absorption spectrum of a diluted solution, which was obtained by adding ethanol to **S** and adjusting the Ti^4+^ concentration to 0.01 mmol·g^−1^, were measured in the wavelength region of 200–1100 nm using a U-2800 spectrophotometer (Hitachi, Tokyo, Japan) in the double beam mode. Ethanol was used as the reference. A 1-mm light-path-length cell made of quartz glass was used to measure the absorption spectrum. The transmittance spectra of **F_0_** and **F_x_** were measured in the same wavelength region by using an identical spectrophotometer in the double beam mode, and air was used as the reference.

The refractive indices of **F_x_** were measured using an ellipsometer MARY-102 (Five Lab, Kawakuchi, Japan). The measurements were performed using light of wavelength 632.8 nm and incident at an angle of 70.07°. The refractive index of each **F_x_** was measured three times at 14 points on the films, and the averaged value was obtained. The reference refractive index used in the measurement was that of a crystal of anatase (= 2.52).

#### 2.4.2. X-Ray Diffraction (XRD) Patterns of **F_0_** and **F_x_**

The X-ray diffraction (XRD) patterns of **F_0_** and **F_x_** were measured using a SmartLab X-ray diffractometer (Rigaku, Tokyo, Japan), with Cu-*K*α rays generated at 45 kV and 200 mA. Parallel beam optics with an incident angle of 0.3° in the 2θ range of 10–80° were used in each measurement.

#### 2.4.3. Chemical Characterization of **F_0_** and **F_x_**

The Fourier-transformed infrared spectra (FT-IR) of **F_0_**, **F_1_**, **F_2_**, **F_4_**, **F_8_**, and **F_16_** were measured by using a FT-IR spectrometer (FT-IR-4600 (JASCO, Tokyo, Japan)) in the wavelength range from 100 to 4000 cm^−1^. X-ray photoelectron spectroscopy (XPS, JEOL, Tokyo, Japan) was used to analyze the chemical component of **F_4_**. A Phi Quantum 2000 Scanning ESCA Microprobe (JEOL, Tokyo, Japan) with a focused monochromatic Al-*K*α X-ray (1486.6 eV) source was employed to evaluate the states and amounts of the five elements Ti, O, C, N, and Si. The chemical shift data were charge-referenced to the center of the C–C peak at 284.5 eV. The resolution was 0.1 eV in each measurement.

#### 2.4.4. Surface Morphology and Film Thickness of **F_x_**

The surface morphology of **F_x_** was observed using a field emission scanning electron microscope (FE-SEM) (JSM-6701F, JEOL, Tokyo, Japan). To measure the film thickness, the cross-sectional image was obtained using this instrument. Prior to the FE-SEM observation, the samples were sputtered with Au by using Quick Coater (SC-701MkII ECO, Sanyu Electron, Tokyo, Japan) for 1 min in order to increase the electrical conductivity of the sample surface.

#### 2.4.5. Pencil Scratching Test for Adhesion Strength of **F_x_**

The adhesion strength of **F_x_** were evaluated using the pencil-scratching method, according to JIS no. K 5600-5-4. Pencils (Mitsubishi, UNI, Tokyo, Japan) with different hardness of pencil from 6B to 9H were used. Each pencil in contact with the samples was shifted 5 times, and the robustness against scratching was observed.

#### 2.4.6. Photo-Induced Hydrophilicity of **F_x_**

The photo-induced hydrophilicity of **F_x_** and the quartz glass substrate was evaluated by measuring the contact angle of a 1.0-μL water droplet placed perpendicularly on the sample surface. The measurement was performed with a contact angle meter (FACE, Kyowa Interface Science, Niiza, Japan). Prior to measurement, all the samples were kept in a dark condition at room temperature for approximately 12 h. Then, the sample surface was irradiated with UV light of wavelength and intensity identical to those in the aforementioned coating procedure, for 10 min at 25°C and 30–40% humidity. After keeping these UV-irradiated samples again in a dark condition for 0, 1, 2, and 4 h, the contact angle of a water droplet placed on the surface was measured in each case. The contact angles of the samples, whose contact angles had been examined once after being kept in a dark condition for 4 h, were again measured after the UV irradiation for 10 min.

## 3. Results

### 3.1. Absorption Spectrum of **S**

**Figure 1:**
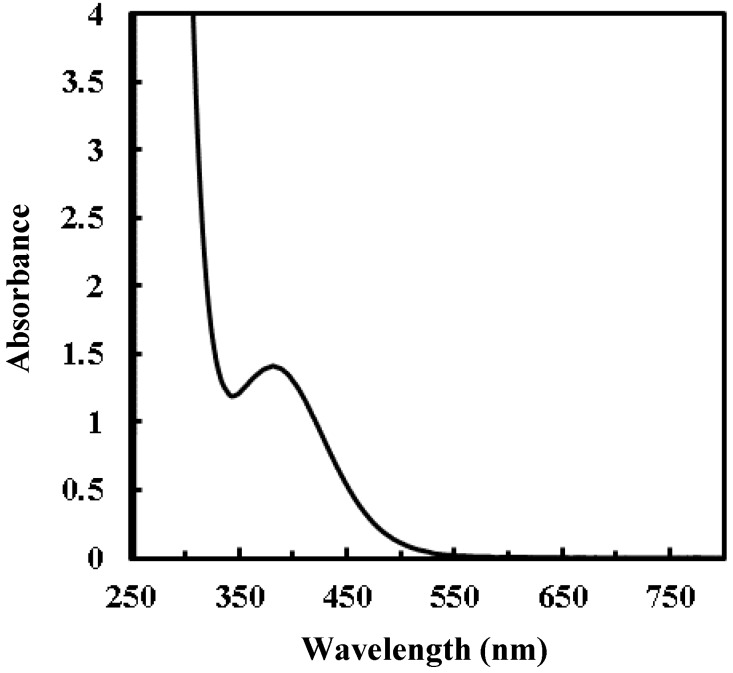
Absorption spectra of diluted solution (Ti^4+^ concentration of 0.01 mmol·g^−1^) of precursor solution **S**. Ethanol was used as the reference.

Figure 1 shows the absorption spectrum of the diluted solution of the precursor solution **S**. A characteristic absorption was observed in the range from 350 to 550 nm with a peak center at 377 nm; moreover, a substantially strong absorption in the UV region was also observed.

### 3.2. Optical Properties of Precursor Film **F_0_** and UV-Irradiated Films **F_x_**

**Figure 2:**
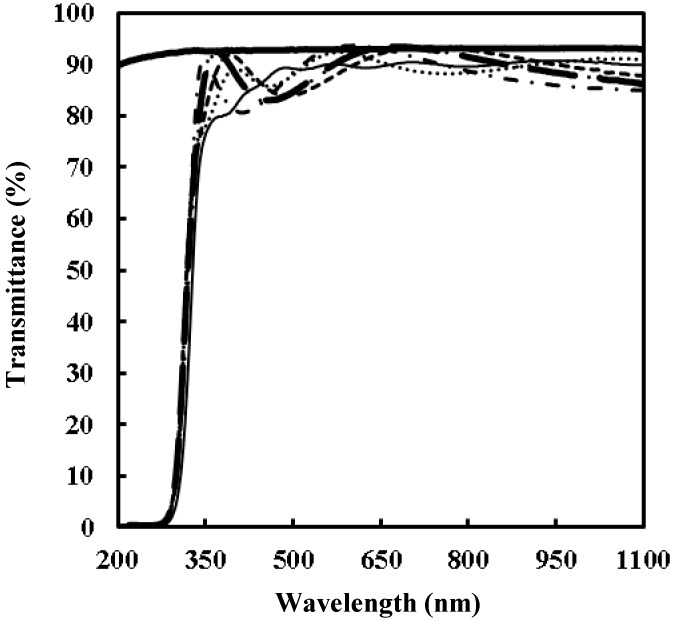
Transmittance spectra of precursor film **F_0_**, ultraviolet (UV)-irradiated films **F_x_**, and quartz glass substrate applied. Air was used as the reference. The lines represent quartz glass substrate (**━**), **F_0_** (—), **F_1_** (…), **F_2_** (---), **F_4_** (**- -**), **F_8_** (-⋅-), and **F_16_** (-⋅⋅-).

Figure 2 presents the transmittance spectra of the precursor film **F_0_** and UV-irradiated films **F_x_** together with that of the quartz glass substrate applied. All the films **F_0_** and **F_x_** exhibited transmittance higher than 80% in the visible region and no transparency in the UV light region of wavelength shorter than approximately 275 nm. Table 1 summarizes the refractive indices of **F_x_**.

### 3.3. XRD Patterns of **F_0_** and **F_x_**

**Figure 3:**
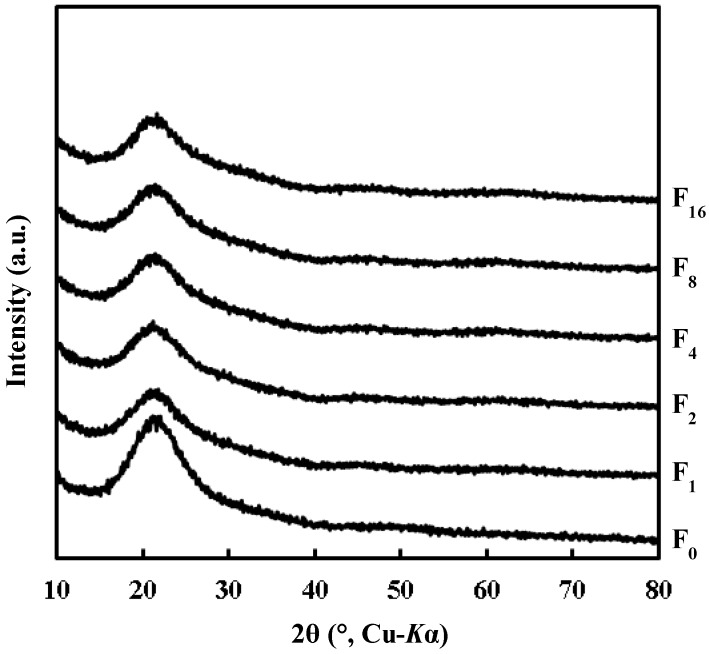
X-ray diffraction (XRD) patterns of precursor film **F_0_** and UV-irradiated films **F_x_** on quartz glass substrate.

Figure 3 shows the XRD patterns of **F_0_** and **F_x_**. These XRD patterns indicate that the resultant thin films on the quartz glass substrate are amorphous because only a hollow band was observed in the 2θ range of 15–30°.

### 3.4. Fourier Transform-Infrared (FT-IR) Spectra of **F_0_** and UV-Irradiated Films **F_x_**, and Quartz Glass Substrate

**Figure 4:**
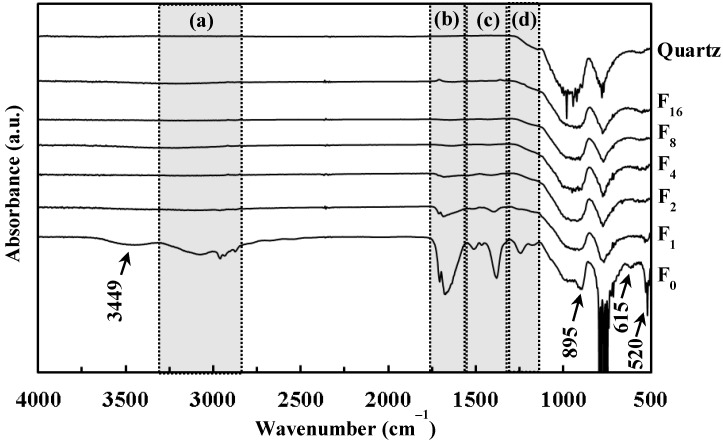
Fourier transform-infrared (FT-IR) spectra of **F_0_**, **F_1_**, **F_2_**, **F_4_**, **F_8_**, **F_16_**, and quartz glass substrate. The peaks in each dotted region correspond to the (**a**) C–H stretching mode of CH_2_/CH_3_ groups, (**b**) C=O stretching mode, (**c**) C–H bending mode of CH_2_/CH_3_ groups, and (**d**) C–O stretching mode, respectively. The peaks of 895, 615, are 520 cm^−1^ are assignable to the O–O stretching of side-on peroxo group bonded to Ti(IV) ion (Ti–O_2_^2−^), to the Ti–O_2_^2−^ symmetric stretching mode, and to the Ti–O_2_^2−^ asymmetric stretching mode, respectively.

Figure 4 shows the FT-IR spectra of **F_0_**, **F_1_**, **F_2_**, **F_4_**, **F_8_**, **F_16_**, and the quartz glass substrate. The weak and broad absorption band observed at approximately 3449 cm^−1^ in the spectrum of **F_0_** corresponds to chemisorbed water [17]. The region (a) involving weak and broad absorptions of 2875, 2934, 2960, and 3071 cm^−1^ correspond to the C–H stretching mode of CH_2_/CH_3_ groups [18,19,20]. The region (b) with peaks in the range 1675–1707 cm^−1^ are assignable to the C=O stretching mode [21]. Three peaks of 1382, 1467, and 1513 cm^−1^ observed in the region (c) are assignable to the C–H bending mode of CH_2_/CH_3_ groups [22,23,24]. Two peaks of 1246 and 1179 cm^−1^ in the region (d) represent the C–O stretching mode [23,25]. The peaks observed at 895, 615, and 520 cm^−1^ are assignable to the O–O stretching of side-on peroxo group bonded to Ti(IV) ion (Ti–O_2_^2−^), to the Ti–O_2_^2−^ symmetric stretching mode, and to the Ti–O_2_^2−^ asymmetric stretching mode, respectively [26,27,28]. Additionally, the broad and weak peaks in the (b) and (c) region were observed from **F_1_** and **F_2_**, while a weak peak at 520 cm^−1^ was observed from **F_1_**.

### 3.5. X-Ray Photoelectron Spectroscopy (XPS) Spectra of UV-Irradiated Film **F_4_**

**Figure 5:**
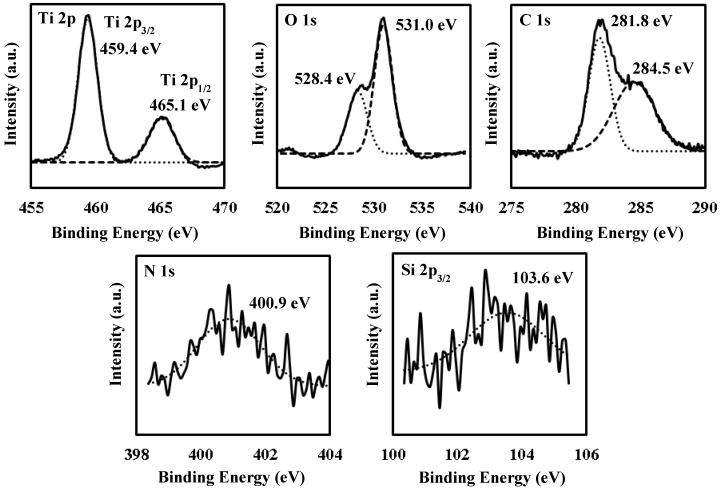
X-ray photoelectron spectroscopy (XPS) spectra of Ti 2p, O 1s, C 1s, N 1s, and Si 2p of surface of **F_4_**. The solid lines indicate the original data of XPS. The dashed and doted curves indicate theoretically fitted curves by assuming Gaussian distribution.

Figure 5 shows the XPS spectra of **F_4_**. Five binding energy ranges corresponding to the Ti 2p, O 1s, C 1s, N 1s, and Si 2p orbitals are presented. The two binding energies of 459.4 and 465.1 eV are assignable to Ti 2p_3/2_ and 2p_1/2_ orbital electrons, respectively, bonded to oxygen (Ti–O) [29,30]. The two binding energies of 528.4 and 531.0 eV can be assigned to the O 1s orbital electrons linked to titanium (O–Ti) and hydrogen (O–H) in the hydroxy group, respectively [31,32]. The two binding energies of 281.8 and 284.5 eV correspond to the C 1s orbital electrons associated with titanium (Ti–C, substitutional carbon of Titania) and carbon (C–C), respectively [33,34]. A binding energy of 400.9 eV corresponds to that of chemisorbed nitrogen [29,35]. The binding energy of 103.6 eV is assignable to the Si 2p orbital electrons bonded to oxygen (SiO_2_) [36]. The relative proportions of the five elements, Ti, O, C, N, and Si calculated from each peak area and sensitivity are 12.0, 53.8, 32.5, 0.9, and 0.9%, respectively. The calculated O/Ti ratio of the Ti–O bond, to which a binding energy of 528.4 eV can be assigned, is approximately 1.53.

### 3.6. Surface Morphology, Film Thickness, and Adhesion Strength of UV-Irradiated Films **F_x_**

**Figure 6:**
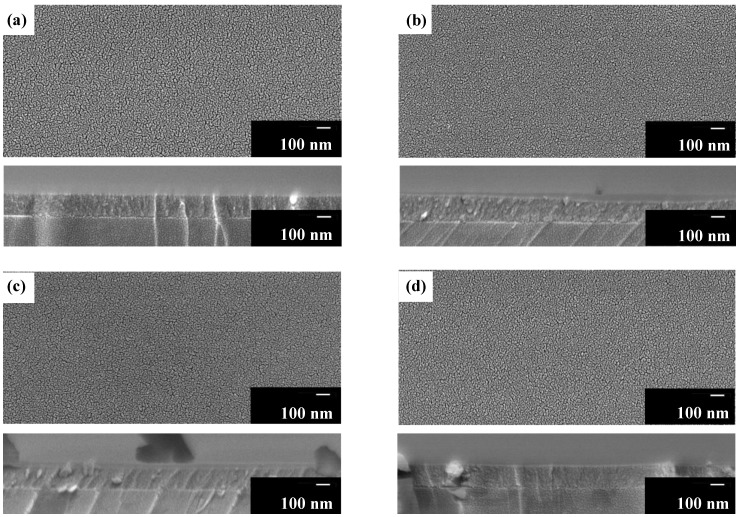
Scanning electron microscope (SEM) images of top and cross-sectional views of (**a**) **F_2_**, (**b**) **F_4_**, (**c**) **F_8_**, and (**d**) **F_16_.** The thicknesses of **F_2_**, **F_4_**, **F_8_**, and **F_16_** are approximately 170, 170, 160, and 160 nm, respectively.

Figure 6 shows the surface morphology and cross-sectional images of **F_x_** (x ≥ 2). The smooth surfaces of **F_x_** indicate that the particles, ca. 10–20 nm in size, are well-connected with neither apparent crack nor pinhole. From the cross-sectional images of **F_x_**, the interface between the film and substrate can be clearly discriminated; moreover, film thicknesses of **F_2_**, **F_4_**, **F_8_**, and **F_16_** were determined as approximately 170, 170, 160, and 160 nm, respectively.

The adhesion strength of **F_1_** is F, whereas those of **F_2_**, **F_4_**, **F_8_**, and **F_16_** are equal to 6H.

### 3.7. Contact Angles of Water Droplet on UV-Irradiated Films **F_x_**

Table 2 summarizes the contact angles of a water droplet on **F_x_**, together with the photographic images captured from the side, when **F_x_** were photo-induced by UV-irradiation for 10 min after being kept for 0, 1, 2, and 4 h in a dark condition. The corresponding values prior to photo-induction and with the quartz glass substrate are also presented in the Table as reference. The contact angle for **F_1_** was not measured because numerous minute cracks appeared on its surface immediately after the placement of a water droplet for measurement.

All the contact angles on **F_x_** prior to the photo-induction by UV-irradiation for 10 min are larger than those of the quartz glass substrate. However, the severe decrease in the values was observed by photo-inducing **F_x_**, although the quartz glass substrate did not exhibit a significant difference. By photo-inducing **F_x_** through UV irradiation for 10 min, all of them exhibited contact angles smaller than 4°; this indicates the photo-induced super-hydrophilicity. The extent of decrease in the contact angles evidently depend on both the light-shielding duration in the dark condition after photo-induction for 10 min and the exposure time to UV light for fabricating the films. The contact angles of the samples, whose contact angles had once been examined after being kept in a dark condition for 4 h, were also smaller than 4°. It was thus clarified that the super-hydrophilicity of **F_x_** can be photo-induced repeatedly by UV-irradiation for 10 min, even after the hydrophilic level has once been lowered by being kept in a dark condition for 4 h.

## 4. Discussion

### 4.1. Precursor Film Involving Ti(IV) Complex of Oxalato and Peroxo Ligands with Butylamine

In this present study, the dried precursor film **F_0_** was prepared on a quartz glass substrate by spin-coating the precursor solution **S** involving a Ti(IV) complex of oxalato and peroxo ligands. The Ti(IV) complex salt can be dissolved in ethanol according to the typical concept of MPM, in the presence of alkylammonium ion as a counter cation. The absorption spectrum of a diluted solution of **S** indicated that the dissolved Ti(IV) complex ion in the ethanol solution, which absorption peak position at 364 nm is comparable to those of the reported Ti–H_2_O_2_-complexes, has a coordination bond between Ti(IV) and O_2_^2^^−^ ions (Figure 1) [37]. The FT-IR spectrum of **F_0_** reveal the presence of side-on type Ti–O_2_^2^^−^ bond, and the characteristic peak owing to the coordination bond between peroxo ligand and Ti(IV) ion can be also observed at 377 nm in the transmittance spectrum of **F_0_** (Figure 2 and Figure 4). Furthermore, the dried precursor film involves the chemical bonds of C–O, C=O, and CH_2_/CH_3_ groups, as exhibited by the FT-IR spectrum (Figure 4). These results reveal the presence of oxalato and peroxo ligands and butylamine moiety in **F_0_**. It was thus demonstrated that the dried precursor film **F_0_** involving the identical solute consisted of the Ti(IV) complex salt in the precursor solution. 

The precursor film **F_0_** involving the Ti(IV) complex salt can be dissolved straightforwardly in both ethanol and water, as expected. Therefore, the precursor film **F_0_** itself is not effective as a hydrophilic material on glass substrates. However, this solubility of the precursors is an advantage of the MPM because in the event that the quality of the precursor films is not satisfactory owing to foreign matters on the substrate, the substrates can be recovered prior to the subsequent step. Therefore, it is important to note that the MPM could produce the photo-induced super-hydrophilic thin film from the precursor film by using a weak UV irradiation under ambient conditions (vide supra). 

### 4.2. Conversion of Precursor Film Involving Ti(IV) Complex Salt to Amorphous Thin Films by Irradiating with Weak UV Light

The weak UV light of 4 mW·cm^−^^2^ at 254 nm was used both in the conversion from the spin-coated precursor film **F_0_** consisting of Ti(IV) complex salt and in the photo-induction of hydrophilicity at the thin-film surface.

The disappearance of the corresponding peaks from the FT-IR spectra of **F_2_** indicates that the peroxo group was gradually removed from the Ti(IV) complex salt in **F_0_** by the UV irradiation for 2 h. In addition, the peaks owing to both oxalato and peroxo ligands vanished after UV irradiation of the precursor film for longer duration, as shown in the spectra of **F_4_**, **F_8_** and **F_16_** (Figure 4). It is considered that these ligands, originally involved in the precursor film, were removed stepwise by the UV irradiation at room temperature owing to the strong absorption assignable to the CT transition band in the UV light region (Figure 1). In addition, a solid film was not obtained in the case of 365 nm UV irradiation to the precursor film for 16 h (see Section 2.3). It is thus indicated that the UV light at 365 nm was not efficient to fabricate within 16 h, while the UV light at 254 nm fabricate a solid film with 4 h.

In the XPS spectrum assignable to the Ti 2p orbital of the **F_4_** surface, the Ti^4+^–O bond was observed in the TiO_2_ skeleton (Figure 5). However, it was clarified by XRD measurement that all the UV-irradiated films were amorphous (Figure 3). These results indicate that the Ti(IV) complex salt involved in the precursor film decomposed to form a non-crystalline TiO_x_ (x = 1.53) thin film at room temperature, where the x value was determined by the XPS measurement (see Section 3.5).

In the case of the crystalline TiO_2_ thin film formation, Hattori et al. reported the crystalline TiO_2_ thin film on a SnO_2_ coated glass substrate formed by irradiation with UV light of wavelength center 350 nm from a high-pressure mercury lamp. A gel-film prepared by mixing Ti(OC_4_H_9_)_4_ and benzoylacetone in methanol was UV-irradiated and heat treated at 500 °C [38]. Li et al. reported the crystalline TiO_2_ thin film fabricated by coating TiO_2_ particles with *N*-methyl-2-pyrrolidone and methanol dispersion on silicon, glass, and poly(ethylene terephthalate) substrates and UV- irradiating (wavelength of 264 nm with intensity of 4.5 mW·cm^−2^) the film at room temperature or 65 °C to degrade the organic components [39]. Zou et al. reported an amorphous TiO_2_ thin film on Pt/Si substrate from a solution involving a mixture of tetrabutyl titanate, acetylacetone, acetic acid, and 2-methoxyethanol. The thin film was fabricated by UV-irradiation from a low-pressure mercury lamp (wavelengths of 185 and 254 nm and intensity of 12.5–13.5 mW·cm^−^^2^), of a spin-coated film for 5 h at room temperature after it had been heat treated at 150 °C for 3 min, while the generation of ozone during the UV irradiation was involved [40].

This present work demonstrates that the precursor film involving Ti(IV) complex could be converted to an unprecedented amorphous thin film by using a weak UV irradiation from an inexpensive germicidal lamp, at room temperature under a non-ozone environment.

### 4.3. Optical and Mechanical Properties of the Amorphous Thin Films

The thin films obtained by UV irradiation for over 1 h exhibit high transmittance of over 80% in the visible region (Figure 2). The refractive indices of **F_2_**, **F_4_**, **F_8_**, and **F_16_** UV-irradiated for over 2 h are almost identical (Table 1), and the values 1.78–1.79 are comparable to those of dense flint glass and are significantly smaller than those of anatase TiO_2_ of 2.56 [41]. In the case of **F_1_**, the corresponding value 1.70 is smaller than those for the other thin films, indicating lower reflectivity. These results indicate that the photo-chemical reaction of the precursor Ti(IV) complex salt was almost completed by the UV irradiation for 4 h of the precursor film **F_0_**. The aforementioned FT-IR spectra indicate that the UV-irradiation for 4 h is adequate to degrade and remove the ligands in these thin films. It was also revealed that the further UV irradiation can contribute toward densifying, stabilizing, and mechanically strengthening the amorphous thin films.

With regard to polycrystalline transparent nanotube films of hydrogen titanate and titania (anatase) nanotube films on Pyrex glass or silicon wafer substrates, Miyauchi et al. reported the fabrication by layer-by-layer self-assembly and by annealing titanate nanotube films at 673 K in air, respectively [41]. Both the types of film exhibited an optically low reflectivity (refractive index of 1.7) and a high transparency compared to polycrystalline anatase thin films. The optical properties of the nanotube films are owing to their nano-structural porosity. In order to prepare the characteristic morphologies, a multi-step and complicated process involving a chemical reaction in a strongly basic solution for 40 h, the application of a 200-W Hg–Xe lamp for 20 h to decompose the added organic polymers, was necessary. However, in the present study, an identical level of refractive index could be obtained from the amorphous thin films without modifying the dense film structure, as demonstrated by the FE-SEM observations (Figure 6).

### 4.4. Photo-Induced Super-Hydrophilicity of the Amorphous Thin Films

The UV-irradiated films of **F_2_**, **F_4_**, **F_8_**, and **F_16_** exhibit super-hydrophilicity, and the contact angles decrease from over 30° to less than 4° after UV irradiation of **F_x_** for 10 min (Table 2). The photo-induced super-hydrophilicity of **F_x_** with contact angles smaller than 4° was demonstrated by UV-irradiating the films for 10 min even after the hydrophilic level of the thin films had been lowered by being kept in a dark condition for 4 h. Thus, the behaviors of the photo-induced hydrophilicity of these amorphous thin films are identical to those of crystalline TiO_2_ [8,42].

It has been reported that the contact angle of the polycrystalline anatase-type TiO_2_ thin film did not decrease below 5° even after super-hydrophilicity was induced by UV irradiation [41,43]. The contact angles lower than 4° observed in the present work is likely to be related to the surface roughness of the resultant films; this is because the hydrophilicity of a film surface can be enhanced by the surface roughness [41]. It can be easy to accept that the removal species from the degraded ligands occurs during the photo-chemical reaction through tiny cracks between the well-connected grains formed in these thin films; this is evident from the scanning electron microscope (SEM) images of **F_2_**, **F_4_**, **F_8_**, and **F_16_** (Figure 6). This likelihood is consistent with the dependency of the contact angles on the duration of UV irradiation.

Gao et al. reported the amorphous TiO_2_ thin film deposited by soaking *p*-Si substrate pre-coated with a self-assembled monolayer in an aqueous solution; it was prepared by mixing H_2_TiO_3_, H_2_O_2_ (30% in H_2_O), and ammonia (25% in H_2_O) in water, with a soaking time of 120 h at room temperature. The films reveal that the contact angles were changed from 34° to below 5° after 5 min of UV irradiation (wavelength centered at 184.9 and 253.7 nm and of intensity 1.8 mW·cm^−2^), together with the achievement of super-hydrophilicity through UV irradiation of the film for 1 min, after the loss of hydrophilicity [44]. Vrakatseli et al. reported an amorphous TiO_x_ film on a glass and polymeric substrate fabricated by spin-coating a solution mixed with titanium isopropoxide, isopropanol, HNO_3_, and H_2_O and dried for over 24 h at room temperature in the dark. The resultant film exhibits a contact angle lower than 5° after UV-irradiation (wavelength of 254 nm and intensity of 8 mW·cm^−2^) for 7 min [45]. In this work applying UV irradiation to fabricate the thin films from a precursor involving a Ti(IV) complex salt, a stable amorphous thin film could be obtained by a short UV-irradiation time of 4 h, even at room temperature; moreover, the functionality of photo-induced super-hydrophilicity with contact angle smaller than 4° are achievable.

## 5. Conclusions

The precursor film involving a Ti(IV) complex of oxalato and peroxo ligands could be converted to an amorphous solid thin film by applying a low-power germicidal lamp having an irradiation peak at 254 nm, for 4–16 h under humidity of 40–60% at room temperature. In this work, metal complex with oxalate and peroxo ligands based on MPM was effectively degraded by the UV-irradiation. The fabricated 160–170-nm amorphous thin films exhibited a high transparency of 80% in the visible region, relatively low refractive indices of 1.78–1.79 and photo-induced super-hydrophilicity with a contact angle lower than 4°, compared to the crystalline anatase.

A comparison of this work with other works indicated the advantages of low cost, low temperature, and a simple process for functional amorphous thin-film coating, whereby the functionality can be conveniently obtained by using only a solution-based coating of MPM with 4 h of UV irradiation from a germicidal lamp of low intensity at room temperature, under a non-ozone environment.

## Figures and Tables

**Table 1:** Refractive indices of UV-irradiated films **F_x_**.

Sample	F_1_	F_2_	F_4_	F_8_	F_16_
**Refractive index**	1.70 ± 0.01	1.79 ± 0.01	1.78 ± 0.01	1.79 ± 0.01	1.79 ± 0.01

**Table 2:**
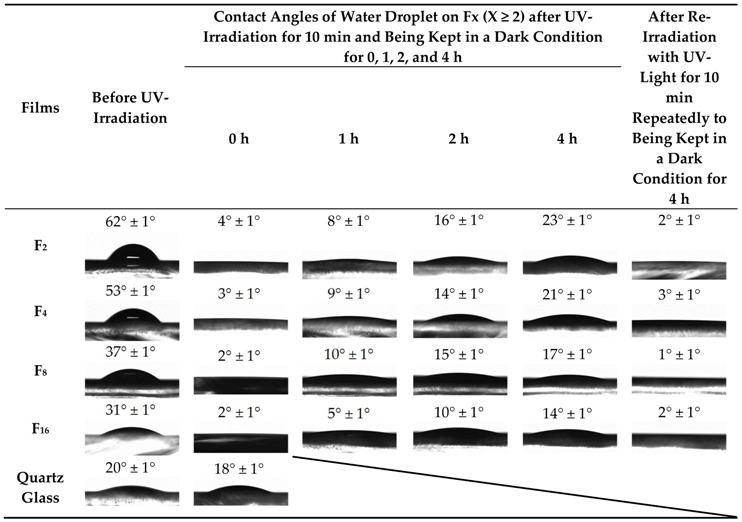
Contact angles of a water droplet on **F_x_** (x ≥ 2) prior to and after photo-induction by UV irradiation for 10 min, together with the photographic images captured from the side and those on the quartz glass substrate. The measurements were performed after the samples were kept for 0, 1, 2, and 4 h in a dark condition. The contact angles of the photo-induced samples (whose contact angles were once examined after being kept in a dark condition for 4 h) subsequent to the second UV-irradiation for 10 min are also presented.

## References

[1] Fateh R., Dillert R., Bahnemann D. (2014). Self-cleaning properties, mechanical stability, and adhesion strength of transparent photocatalytic TiO_2_-ZnO coatings on polycarbonate. ACS Appl. Mater. Interfaces.

[2] Latthe S., Liu S., Terashima C., Nakata K., Fujishima A. (2014). Transparent, Adherent, and Photocatalytic SiO_2_-TiO_2_ Coatings on Polycarbonate for Self-Cleaning Applications. Coatings.

[3] Adachi T., Latthe S.S., Gosavi S.W., Roy N., Suzuki N., Ikari H., Kato K., Katsumata K.I., Nakata K., Furudate M. (2018). Photocatalytic, superhydrophilic, self-cleaning TiO_2_ coating on cheap, light-weight, flexible polycarbonate substrates. Appl. Surf. Sci..

[4] Perkas N., Gedanken A. Coatings of polymers with TiO_2_ nanoparticles by sonochemical method. Proceedings of the 2011 NSTI Nanotechnology Conference and Expo, NSTI-Nanotech 2011.

[5] Miyauchi M. (2008). Photocatalysis and photoinduced hydrophilicity of WO_3_ thin films with underlying Pt nanoparticles. Phys. Chem. Chem. Phys..

[6] Bashir S., Bashir M., Casadevall X., Rees J.M., Zimmerman W.B. (2015). Hydrophilic Surface Modification of PDMS Microchannel for O/W and W/O/W Emulsions. Micromachines.

[7] Schneider J., Matsuoka M., Takeuchi M., Zhang J., Horiuchi Y., Anpo M., Bahnemann D.W. (2014). Understanding TiO_2_ photocatalysis: Mechanisms and materials. Chem. Rev..

[8] Nakata K., Fujishima A. (2012). TiO_2_ photocatalysis: Design and applications. J. Photochem. Photobiol. C Photochem. Rev..

[9] Luttrell T., Halpegamage S., Tao J., Kramer A., Sutter E., Batzill M. (2014). Why is anatase a better photocatalyst than rutile?—Model studies on epitaxial TiO_2_ films. Sci. Rep..

[10] Chaiyakun S., Buranawong A., Deelert T., Witit-anun N. (2008). The Influence of Total and Oxygen Partial Pressures on Structure and Hydrophilic Property of TiO_2_ Thin Films Deposited by Reactive DC Magnetron Sputtering. Adv. Mater. Res..

[11] Nagai H., Aoyama S., Hara H., Mochizuki C., Takano I., Baba N., Sato M. (2009). Rutile thin film responsive to visible light and with high UV light sensitivity. J. Mater. Sci..

[12] Nagai H., Sato M., Czerwinski F. (2012). Heat Treatment in Molecular Precursor Method for Fabricating Metal Oxide Thin Films. Heat Treatment—Conventional and Novel Applications.

[13] Nagai H., Sato M., Nikitenkov N. (2017). Molecular Precursor Method for Fabricating *p*-Type Cu_2_O and Metallic Cu Thin Films. Modern Technologies for Creating the Thin-Film Systems and Coatings.

[14] Hishimone P., Nagai H., Morita M., Sakamoto T., Sato M. (2018). Highly-Conductive and Well-Adhered Cu Thin Film Fabricated on Quartz Glass by Heat Treatment of a Precursor Film Obtained Via Spray-Coating of an Aqueous Solution Involving Cu(II) Complexes. Coatings.

[15] Wu H.J., Nagai H., Sato M. (2019). Fabrication of a *p*-type Cu_2_O thin film via UV-irradiation of a patternable molecular-precursor film containing Cu(II) complexes. J. Cryst. Growth.

[16] Nagai H., Mochizuki C., Hara H., Takano I., Sato M. (2008). Enhanced UV-sensitivity of vis-responsive anatase thin films fabricated by using precursor solutions involving Ti complexes. Sol. Energy Mater. Sol. Cells.

[17] Guo X., Navrotsky A. (2018). Hydration dynamics in zeolite A—An X-ray diffraction and infrared spectroscopic study. Microporous Mesoporous Mater..

[18] Sajan D., Joe H., Jayakumar V.S., Zaleski J. (2006). Structural and electronic contributions to hyperpolarizability in methyl p-hydroxy benzoate. J. Mol. Struct..

[19] Pandiarajan S., Umadevi M., Rajaram R.K., Ramakrishnan V. (2005). Infrared and Raman spectroscopic studies of L-valine L-valinium perchlorate monohydrate. Spectrochim. Acta Part A Mol. Biomol. Spectrosc..

[20] Suryanarayanan C., Prasannan A., Hong P.D., Sambathkumar B., Somanathan N. (2014). Variable temperature studies on mesogenic polythiophenes using 2D-IR and WAXS. Mater. Chem. Phys..

[21] Weisz A.D., Garc L., Regazzoni A.E., Blesa M.A. (2002). FTIR study of the adsorption of single pollutants and mixtures of pollutants onto titanium dioxide in water: Oxalic and salicylic acids. Catal. Today.

[22] Kurar S., Rathore D.S., Garg G., Khatri K., Saxena R., Sahu S.K. (2017). Synthesis and Evaluation of Some Benzothiazole Derivatives As Antidiabetic Agents. Int. J. Pharm. Pharm. Sci..

[23] Xu C.H., Sun S.Q., Guo C.Q., Zhou Q., Tao J.X., Noda I. (2006). Multi-steps Infrared Macro-fingerprint Analysis for thermal processing of Fructus viticis. Vib. Spectrosc..

[24] Vogel E., Gbureck A., Kiefer W. (2000). Vibrational spectroscopic studies on the dyes cresyl violet and coumarin 152. J. Mol. Struct..

[25] Saleh T.A., Gupta V.K. (2012). Photo-catalyzed degradation of hazardous dye methyl orange by use of a composite catalyst consisting of multi-walled carbon nanotubes and titanium dioxide. J. Colloid Interface Sci..

[26] Ohno T., Masaki Y., Hirayama S., Matsumura M. (2001). TiO_2_-photocatalyzed epoxidation of 1-Decene by H_2_O_2_ under visible light. J. Catal..

[27] Dakanali M., Kefalas E.T., Raptopoulou C.P., Terzis A., Voyiatzis G., Kyrikou I., Mavromoustakos T., Salifoglou A. (2003). A New Dinuclear Ti(IV)-Peroxo-Citrate Complex from Aqueous Solutions. Synthetic, Structural, and Spectroscopic Studies in Relevance to Aqueous Titanium(IV)-Peroxo-Citrate Speciation. Inorg. Chem..

[28] Sofetis A., Fotopoulou F., Raptopoulou C.P., Zafiropoulos T.F., Perlepes S.P., Klouras N. (2009). Reactions of titanocene dihalides with *N*,*N*′,*N*″-chelates: Preparation, X-ray structure and characterization of bis(chloro){2,6-bis[(3,5-dimethyl)pyrazol-1-yl]pyridine}(η2-peroxo)titanium(IV). Polyhedron.

[29] Xing M., Zhang J., Chen F. (2009). New approaches to prepare nitrogen-doped TiO_2_ photocatalysts and study on their photocatalytic activities in visible light. Appl. Catal. B Environ..

[30] Jedsukontorn T., Saito N. (2018). Photoinduced Glycerol Oxidation over Plasmonic Au and AuM (M = Pt, Pd and Bi) Nanoparticle-Decorated TiO_2_ Photocatalysts. Nanomaterials.

[31] Ren D., Cui X., Shen J.I.E., Zhang Q.U.N., Yang X., Zhang Z., Ming L.U. (2004). Study on the Superhydrophilicity of the SiO_2_-TiO_2_ Thin Films Prepared by Sol-Gel Method at Room Temperature. J. Sol-Gel Sci. Technol..

[32] Baghriche O., Rtimi S., Pulgarin C., Roussel C., Kiwi J. (2013). RF-plasma pretreatment of surfaces leading to TiO_2_ coatings with improved optical absorption and OH-radical production. Appl. Catal. B Environ..

[33] Chen X., Glans P., Qiu X., Dayal S., Jennings W.D., Smith K.E., Burda C., Guo J. (2008). X-ray spectroscopic study of the electronic structure of visible-light. J. Electron Spectrosc. Relat. Phenom..

[34] Yang J., Bai H., Tan X., Lian J. (2006). IR and XPS investigation of visible-light photocatalysis-Nitrogen-carbon-doped TiO_2_ film. Appl. Surf. Sci..

[35] Xie Y., Li Y., Zhao X. (2007). Low-temperature preparation and visible-light-induced catalytic activity of anatase F-N-codoped TiO_2_. J. Mol. Catal. A Chem..

[36] Zakaznova-Herzog V.P., Nesbitt H.W., Bancroft G.M., Tse J.S., Gao X., Skinner W. (2005). High-resolution valence-band XPS spectra of the nonconductors quartz and olivine. Phys. Rev. B.

[37] Mehra M.C., Easter A. (1997). Colorimetric Determination of Titanium as Peroxomethylenediaminetetracarboxylate. Asian J. Chem..

[38] Hattori A., Tokihisa Y., Tada H., Tohge N., Ito S., Hongo K., Shiratsuchi R., Nogami G. (2001). Patterning Effect of a Sol-Gel TiO_2_ Overlayer on the Photocatalytic Activity of a TiO_2_/SnO_2_ Bilayer-Type Photocatalyst. J. Sol-Gel Sci. Technol..

[39] Li C., Colella N.S., Watkins J.J. (2015). Low-Temperature Fabrication of Mesoporous Titanium Dioxide Thin Films with Tunable Refractive Indices for One-Dimensional Photonic Crystals and Sensors on Rigid and Flexible Substrates. ACS Appl. Mater. Interfaces.

[40] Zou L., Hu W., Xie W., Chen R., Qin N., Li B., Bao D. (2014). Applied Surface Science Excellent resistive switching property and physical mechanism of amorphous TiO_2_ thin films fabricated by a low-temperature photochemical solution deposition method. Appl. Surf. Sci..

[41] Miyauchi M., Tokudome H. (2006). Low-reflective and super-hydrophilic properties of titanate or titania nanotube thin films via layer-by-layer assembly. Thin Solid Film.

[42] Fujishima A., Rao T.N., Tryk D.A. (2000). Titanium dioxide photocatalysis. J. Photochem. Photobiol. C Photochem. Rev..

[43] Eshaghi A., Eshaghi A. (2012). Investigation of superhydrophilic mechanism of titania nano layer thin film-Silica and indium oxide dopant effect. Bull. Mater. Sci..

[44] Gao Y., Masuda Y., Koumoto K. (2004). Light-Excited Superhydrophilicity of Amorphous TiO_2_ Thin Films Deposited in an Aqueous Peroxotitanate Solution. Longmuir.

[45] Vrakatseli V.E., Pagonis E., Amanatides E., Mataras D. (2014). Photoinduced superhydrophilicity of amorphous TiO_x_-like thin films by a simple room temperature sol-gel deposition and atmospheric plasma jet treatment. J. Phys. Conf. Ser..

